# Bayesian optimization of hydrogen plasma treatment in silicon quantum dot multilayer and application to solar cells

**DOI:** 10.1186/s11671-023-03821-9

**Published:** 2023-03-13

**Authors:** Fuga Kumagai, Kazuhiro Gotoh, Satoru Miyamoto, Shinya Kato, Kentaro Kutsukake, Noritaka Usami, Yasuyoshi Kurokawa

**Affiliations:** 1 Department of Materials Process Engineering, Graduate School of Engineering, Nagoya University, Furo-cho, Chikusa-ku, Nagoya, 464-8603 Japan; 2 Department of Electrical and Mechanical Engineering, Nagoya Institute of Technology, Showa-ku, Nagoya, 466-8555 Japan; 3 Center for Advanced Intelligence Project, RIKEN, Tokyo, 103-0027 Japan

**Keywords:** Silicon quantum dot, Solar cell, Bayesian optimization, Dangling bond, Hydrogen plasma

## Abstract

Silicon quantum dot multilayer (Si-QDML) is a promising material for a light absorber of all silicon tandem solar cells due to tunable bandgap energy in a wide range depending on the silicon quantum dot (Si-QD) size, which is possible to overcome the Shockley–Queisser limit. Since solar cell performance is degenerated by carrier recombination through dangling bonds (DBs) in Si-QDML, hydrogen termination of DBs is crucial. Hydrogen plasma treatment (HPT) is one of the methods to introduce hydrogen into Si-QDML. However, HPT has a large number of process parameters. In this study, we employed Bayesian optimization (BO) for the efficient survey of HPT process parameters. Photosensitivity (PS) was adopted as the indicator to be maximized in BO. PS (*σ*_p_/*σ*_d_) was calculated as the ratio of photoconductivity (*σ*_p_) and dark conductivity (*σ*_d_) of Si-QDML, which allowed the evaluation of important electrical characteristics in solar cells easily without fabricating process-intensive devices. 40-period layers for Si-QDML were prepared by plasma-enhanced chemical vapor deposition method and post-annealing onto quartz substrates. Ten samples were prepared by HPT under random conditions as initial data for BO. By repeating calculations and experiments, the PS was successfully improved from 22.7 to 347.2 with a small number of experiments. In addition, Si-QD solar cells were fabricated with optimized HPT process parameters; open-circuit voltage (*V*_OC_) and fill factor (FF) values of 689 mV and 0.67, respectively, were achieved. These values are the highest for this type of device, which were achieved through an unprecedented attempt to combine HPT and BO. These results prove that BO is effective in accelerating the optimization of practical process parameters in a multidimensional parameter space, even for novel indicators such as PS.

**Supplementary Information:**

The online version contains supplementary material available at 10.1186/s11671-023-03821-9.

## Introduction

Over the past few years, many researchers have shown an interest in silicon nanostructures, such as silicon quantum dots (Si-QDs) [1–8], silicon nanowires (SiNWs) [9–14] and so on, to apply to solar cell structure. Since a Si-QD or SiNW embedded in a wide-gap material can confine electrons in zero or one dimension, the bandgap can be tuned in a wide range by controlling their size due to the quantum size effect. Thus, it is possible to apply silicon nanostructure to all silicon tandem solar cells to require light absorbers with different bandgaps, which have possibility to overcome the Shockley–Queisser limit [15]. Widely used single-junction solar cells lose most of the solar spectrum as unavoidable losses such as quantum and transmission losses [16]. Tandem configuration is one of the solutions to reduce these losses and increase theoretical efficiency from 30 to more than 40% [17]. Schygulla et al. reported a two-terminal wafer-bonded III–V//crystalline Si (c-Si) triple-junction solar cell with the efficiency of 35.9% [18, 19]. Although III–V semiconductor is toxic and expensive, silicon is abundant on the earth and non-toxic. Although the bandgap of bulk Si is fixed at 1.12 eV, the quantum size effect enables us to tune the bandgap [20, 21]. We reported that in a theoretical simulation, the bandgap of Si-QDs embedded in amorphous silicon carbide (a-SiC) can be controlled to 1.7 eV by reducing the diameter of Si-QDs to 3 nm [4]. Photoluminescence measurement revealed that the bandgap was changed from 1.1 to 1.6 eV in the diameter range from 10 to 3 nm [1]. The formation method of Si-QDs within a host material is solid phase crystallization as pioneered exemplarily in silicon dioxide (SiO_2_) by Zacharias et al. [22, 23]. It involves depositing a multilayer consisting of alternating layers of silicon-rich and stoichiometric layers of a silicon-based dielectric such as SiO_2_, Si_3_N_4_, or SiC [8, 22–28]. The silicon-rich dielectric phase separates into stoichiometric dielectric and c-Si on thermal annealing [4]:

1 $$\begin{array}{*{20}c} {{\text{Si}}\left( {{\text{O}},{\text{N}},{\text{C}}} \right)_{x} \to \frac{x}{2}{\text{SiO}}_{2} + \left( {1 - \frac{x}{2}} \right){\text{Si}}} \\ \end{array}$$

The size of the Si-QDs can in principle be constrained in one direction by keeping the silicon-rich layers suitably thin and in all other directions by tuning the composition of the silicon-rich layer [3, 29]. Some researchers have tried to fabricate silicon quantum dot solar cell (Si-QDSC) structure using the solid phase crystallization approach. Perez-Wurfl et al. fabricated the p-i-n solar cell structure using silicon quantum dot multilayer (Si-QDML) with SiO_2_ and the open-circuit voltage (*V*_OC_) of 492 mV [30]. The SiO_2_ matrix can reduce dangling bonds of Si-QD surface, leading to a high level of surface passivation of Si-QD [31]. However, short-circuit current density (*J*_SC_) was very poor due to the low tunneling probability of photogenerated carriers, which is caused by the large band offset between c-Si and SiO_2_. To enhance the tunneling probability, we focused on amorphous silicon oxycarbide (a-SiCO_*x*_) and O-deficient amorphous silicon oxide (a-SiO_*y*_) as a barrier material. Yamada et al. achieved a high *V*_OC_ of 518–529 mV in Si-QDSCs using a-SiCO_*x*_ as the matrix material [5, 32]. Akaishi et al. proposed a Si-QDML using a-SiO_*y*_ to improve the short-circuit current (*J*_SC_) by increasing the tunneling probability of photogenerated carriers [33].

To enhance the efficiency of Si-QDSCs, the quality improvement of Si-QDML is necessary. Dangling bonds (DBs) on the surface of Si-QDs form defect levels in the bandgap and promote recombination of minority carriers, which leads to lower solar cell performance. In the Si-QDML preparation process, hydrogen termination of DBs is extremely important. Recently, hydrogen plasma treatment (HPT) has been reported to be an effective method for terminating DBs with hydrogen atoms [1, 2]. In detail, some researchers have reported achieving high passivation effects in Si-QDML with optimized HPT process temperatures or process times [6, 34]. On the other hand, since there is little study about optimizing all HPT process parameters, further improvement of passivation performance can be expected by optimizing all HPT process parameters. Bayesian optimization (BO) was applied to optimize HPT process parameters. Optimizing all HPT process parameters by comprehensive experimentation is enormously time-consuming due to the large number of process parameters. In fact, there were at least six parameters for HPT. BO is a powerful method to optimize multiple parameters based on probabilistic predictions, leading to the reduction of the number of experiments for optimization in a multidimensional parameter space [35]. For example, Miyagawa et al. recently applied BO to the optimization of seven parameters including HPT process parameters in TiO_*x*_/SiO_*y*_/c-Si structures and reported the achievement of a significant improvement in carrier selectivity in just 12 experiment cycles [36]. Therefore, in this study, we attempted to optimize the HPT process parameters efficiently by applying BO to prepare low defect Si-QDML process.

As a quality indicator in Si-QDML, we adopted photosensitivity (PS), which can sensitively reflect the effect of defect quantity and allows the evaluation of important electrical characteristics in solar cells easily without preparing process-intensive solar cells. PS (*σ*_p_/*σ*_d_) is defined as the ratio of photoconductivity (*σ*_p_) and dark conductivity (*σ*_d_), which has been adopted as a quality indicator for thin films by other researchers [37, 38]. In general, *σ*_p_, *σ*_d_, and PS are expressed as follows,

2 $$\begin{array}{*{20}c} {\sigma_{\text{p}} = \frac{e}{d}\eta \mu \tau F\left( {1 - R} \right)\left\{ {1 - \exp \left( { - \alpha d} \right)} \right\} \propto \alpha \eta \mu \tau } \\ \end{array}$$

3 $$\begin{array}{*{20}c} {\sigma_{{\text{d}}} = eN_{{\text{C}}} \mu \exp \left[ { - \left( {E_{{\text{C}}} - E_{{\text{F}}} } \right)/k_{{\text{B}}} T} \right]} \\ \end{array}$$

4 $$\begin{array}{*{20}c} {\frac{{\sigma_{{\text{p}}} }}{{\sigma_{{\text{d}}} }} \propto \frac{\alpha \eta \mu \tau }{{N_{{\text{C}}} \mu \exp \left[ { - \left( {E_{{\text{C}}} - E_{{\text{F}}} } \right)/k_{{\text{B}}} T} \right]}} \propto \frac{\tau }{{\exp \left[ { - \left( {E_{{\text{C}}} - E_{{\text{F}}} } \right)/k_{{\text{B}}} T} \right]}}} \\ \end{array}$$

where *e* is elementary charge, *d* is the thickness of thin films, *α* is the absorption coefficient, *η* is the quantum efficiency, *μ* is the carrier mobility, *τ* is the carrier lifetime, *F* is the incident photon flux, *R* is the surface reflectance, *N*_C_ is the effective density of states in the conduction band, *E*_C_ is the energy level at the conduction band minimum, *E*_F_ is the Fermi level, *k*_B_ is Boltzmann constant and *T* is absolute temperature. Furthermore, by omitting elements which are not changed by light irradiation, this model consists of three elements: *τ*, *E*_C_, and *E*_F_. PS is a quality indicator for the following reasons:

1. *τ* is affected by the amount of hydrogen termination of the DBs.

Since *τ* increases with hydrogen termination of DBs by HPT, high PS is an indicator of high-quality thin films.

2. *E*_F_ is affected by the amount of thermal donor generation due to oxygen defects [39] (see Supplementary information 1).

Since *μ* and *τ* are not high in Si-QD layers, internal electric field is necessary to collect photogenerated carriers in the Si-QD layers. To obtain internal electric field, Si-QD layers should be intrinsic. Thermal donor generation increases carrier concentration and dark conductivity in Si-QDML, leading to the decrease in PS. Therefore, PS is regarded as an indicator of the quality of thin films for p-i-n-type solar cells.

In addition, solar cells were fabricated with Si-QDML prepared according to the above. Therefore, in this study, Si-QDML with oxygen-deficient amorphous silicon oxide was adopted as the absorption layer, and niobium-doped titanium oxide (TNO, TiO_*x*_:Nb) layer was deposited between the Si-QDML layer and the n-type polycrystalline silicon (n^++^-poly-Si) layer [5, 32, 40, 41].

## Experimental methods

### Preparation of Si-QDML

**Fig. 1:**
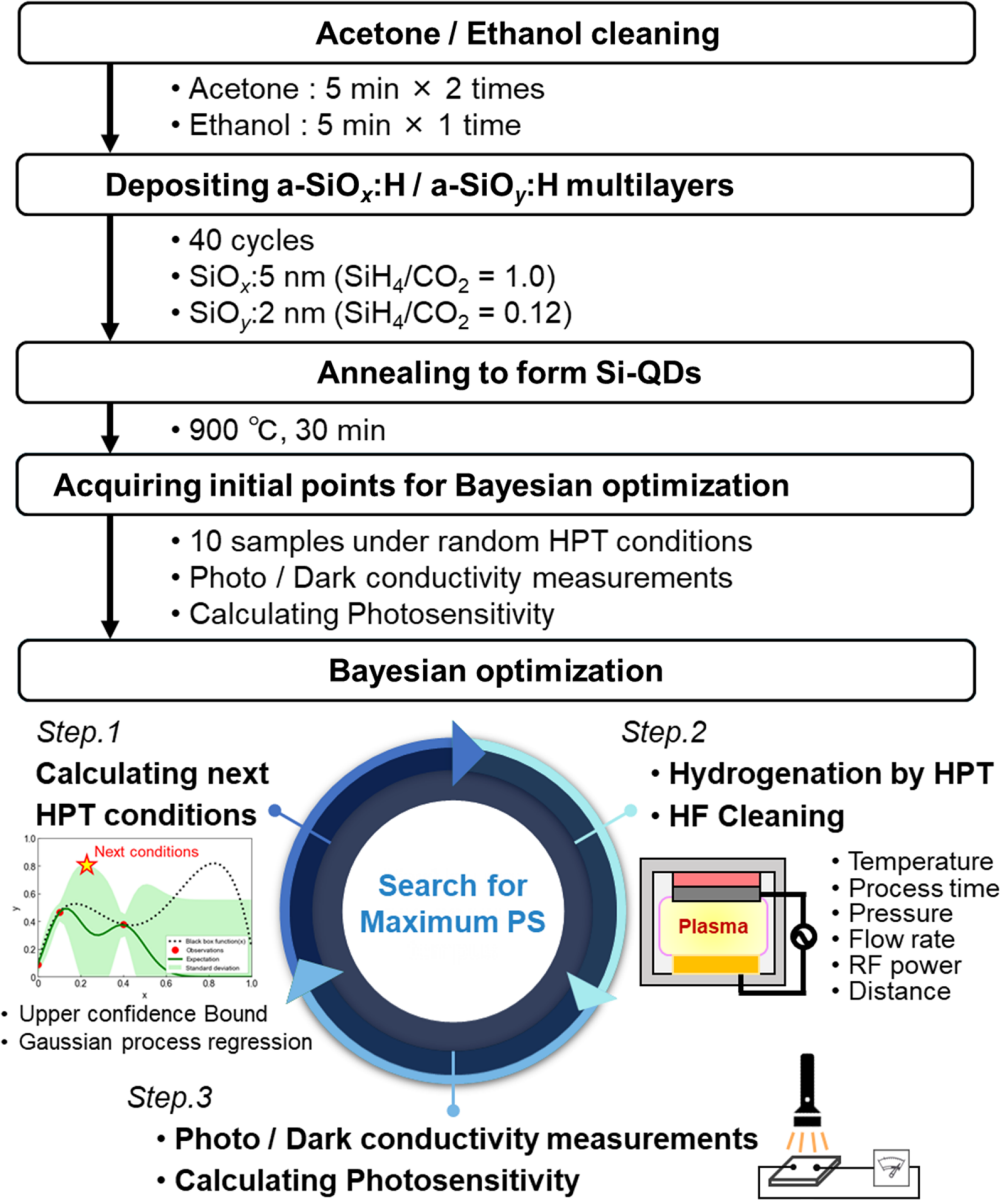
Schematic of preprocessing for BO and optimization cycle of preparation of Si-QDML

The preparation process of Si-QDML applying BO in this study is shown in Fig. 1. A quartz substrate was ultrasonically cleaned with acetone and ethanol before deposition. Hydrogenated amorphous silicon oxide multilayers (a-SiO_*x*_:H/a-SiO_*y*_:H (*x* < *y*)) were prepared on a quartz substrate by plasma-enhanced chemical vapor deposition (PECVD) method (ULVAC, CME-200J). The thicknesses of a-SiO_*x*_:H (Si-rich layer) and the SiO_*y*_:H layer (Barrier layer) were 5 and 2 nm, respectively. The period is 40 cycles (see Supplementary information 2). The SiH_4_/CO_2_ ratios of the Si-rich and barrier layers were 1.0 and 0.12, respectively. The frequency of the plasma supply was 27.12 MHz, the deposition temperature was 195 °C, the chamber pressure was 25 Pa, and the RF power was 32.5 mW/cm^2^. Note that the optical band gaps of the a-SiO_*x*_:H layer, a-SiO_*y*_:H layer, and a-SiO_*x*_:H/a-SiO_*y*_:H (*x* < *y*) multilayer were 1.95, 2.43, and 2.04 eV, respectively (see Supplementary information 3). After deposition, the samples were annealed at 900 °C for 30 min in a forming gas atmosphere (MILA-5050, Advance Science and Technology Corporation). The forming gas composition ratio was N_2_:H_2_ = 97:3, and the flow rate was kept at 0.1 L/min during annealing. At the same time as Si-QDs were formed inside the Si-rich layer by annealing, hydrogen was desorbed from the samples. The HPT was performed in a HTP machine (Katagiri Engineering, Inc.). Six HPT process parameters were optimized by BO: process temperature (*T*_HPT_), process time (*t*_HPT_), H_2_ pressure (*p*_H2_), H_2_ flow rate (*R*_H2_), RF power (*P*_RF_), and electrode distance (*d*). The parameter values were searched within the empirically determined range shown in Table 1.

**Table 1:** Maximum and minimum values of HPT process parameters

	*T*_HPT_ (°C)	*t*_HPT_ (min)	*p*_H2_ (Pa)	*R*_H2_ (sccm)	*P*_RF_ (W)	*d* (mm)
Minimum value	200	1	200	20	280	10
Maximum value	800	60	800	200	500	70

The thickness of the Si-QDML film after HPT was about 200–250 nm. The coplanar Ag electrodes were deposited at an electrode distance of 0.2 mm and an electrode width of 3.4 mm. The applied voltage, the light source, and light intensity for *σ*_p_ measurement were −50 to 50 V, AM1.5G, and 100 mW/cm^2^, respectively. *σ*_p_ and *σ*_d_ were measured and PS was calculated. In general, conductivity of Si-based thin films for solar cells is measured by coplanar electrode configuration, even microcrystalline silicon thin films with structural anisotropy. Therefore, we also adopted the coplanar electrode configuration. Lateral resistance is larger enough than vertical resistance in this coplanar configuration. That is why the conductivity measured by the coplanar electrode configuration is determined by the lateral conductivity. Improvement of lateral photoconductivity suggests the quality improvement of each Si-QD layer. It is presumed that it leads to the improvement of vertical photoconductivity, since photogenerated carriers must pass through each Si-QD layer, when the effect of the barrier layers was ignored. In this case, the photoconductivity measured by coplanar electrode configuration can be used as an index for the improvement of solar cell performance.

### Bayesian optimization

**Fig. 2:**
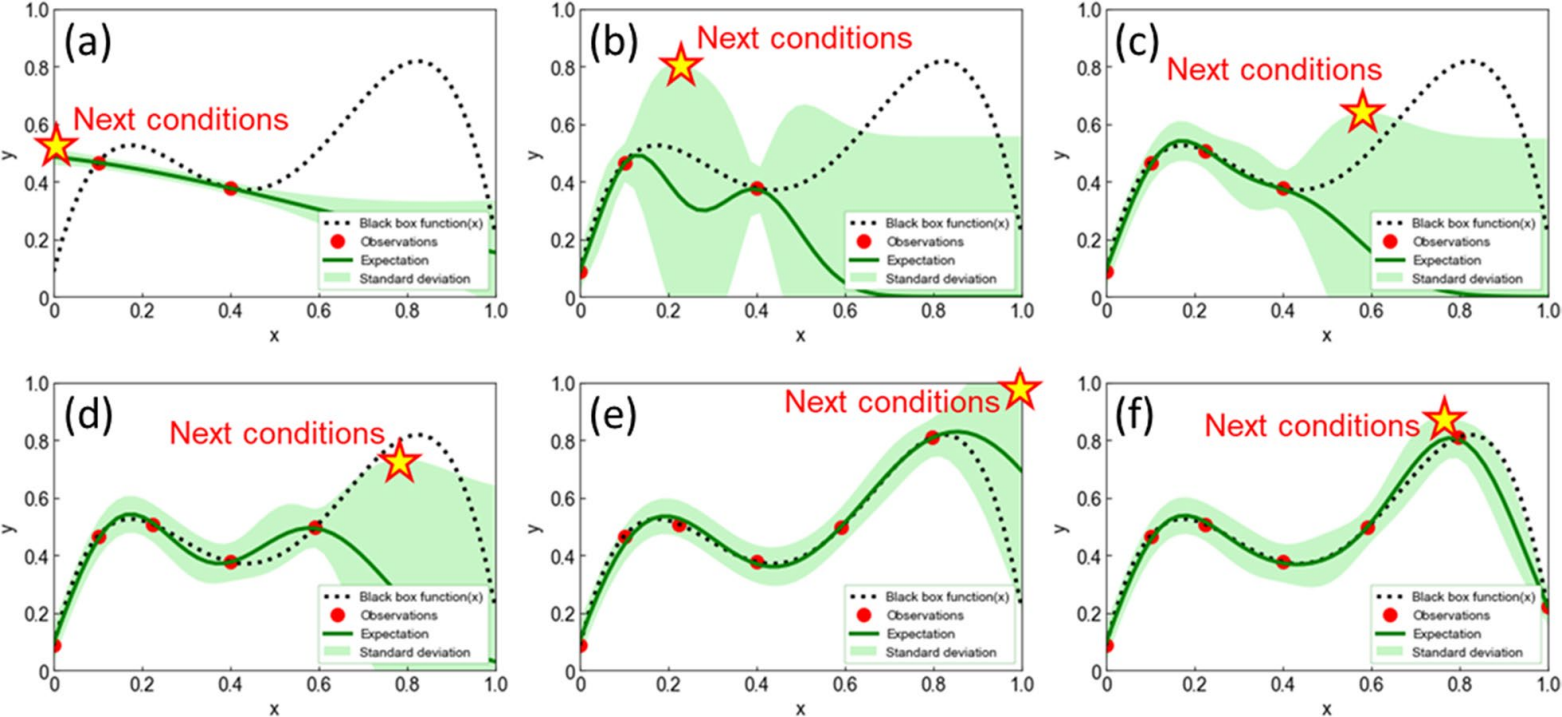
Schematic of BO: GPR results and transitions of optimization by BO based on UCB. The black dotted and red circled markers are the hypothetical black box function and the obtained experimental results, respectively. The green line and light green band indicate the expected value curve and standard deviation, respectively. The star mark means the maximum value of UCB and the next experimental parameters. The prediction model is then updated with the results of the next experimental parameters

Figure 2 shows schematic of BO process. Mathematically, the objective function is expressed as a function $f\left( {\varvec{x}} \right)$ that takes an experimental parameter values ***x*** and returns an objective function value $f\left( {\varvec{x}} \right)$. In particular, when the number of experimental parameters giving the objective function value is small, uncertainty arises in the prediction of the objective function value $f\left( {\varvec{x}} \right)$. To properly handle this prediction uncertainty, the following Gaussian process regression (GPR) was adopted [42].

5 $$\begin{array}{*{20}c} {f\left( {\varvec{x}} \right) \approx N\left( {\mu \left( {\varvec{x}} \right), \sigma^{2} \left( {\varvec{x}} \right)} \right)} \\ \end{array}$$

where $N\left( {\mu \left( {\varvec{x}} \right), \sigma^{2} \left( {\varvec{x}} \right)} \right)$ is normal distribution with the mean $\mu \left( {\varvec{x}} \right)$ and variance $\sigma^{2} \left( {\varvec{x}} \right)$ depending on the input parameter *x*. Meaning, $f\left( {\varvec{x}} \right)$ is estimated from the mean $\mu \left( {\varvec{x}} \right)$, and the variance $\sigma^{2} \left( {\varvec{x}} \right)$ is an indicator to evaluate the uncertainty of $\mu \left( {\varvec{x}} \right)$. The upper confidence bound (UCB), which was the weighted sum of $\mu \left( {\varvec{x}} \right)$ and $\sigma \left( {\varvec{x}} \right)$, was used as the acquisition function $a_{{{\text{UCB}}}} \left( {\varvec{x}} \right)$.

6 $$\begin{array}{*{20}c} {a_{{{\text{UCB}}}} \left( {\varvec{x}} \right) \approx \mu \left( {\varvec{x}} \right) + \kappa \sigma \left( {\varvec{x}} \right)} \\ \end{array}$$

where *κ* is the hyperparameter that determines the balance between the exploration and the exploitation, and $\kappa = 1$ was used in this study. The next HPT process parameters were determined to maximize $a_{{{\text{UCB}}}} \left( {\varvec{x}} \right)$. An example is shown in Fig. 2. HPT was conducted under the determined process parameters, and the PS was measured in the process. The obtained PS and the corresponding HPT process parameters were added to the data set for calculating the next HPT process parameters. In this way, experiments and calculations were performed repeatedly until the next process parameters were calculated to be the same as in the past, where the optimization was complete. The BO process stared with initial 10 experimental results performed under random HTP process conditions. Additionally, 7 experiments performed at the process condition determined by BO.

### Fabrication of solar cell structure

**Fig. 3:**
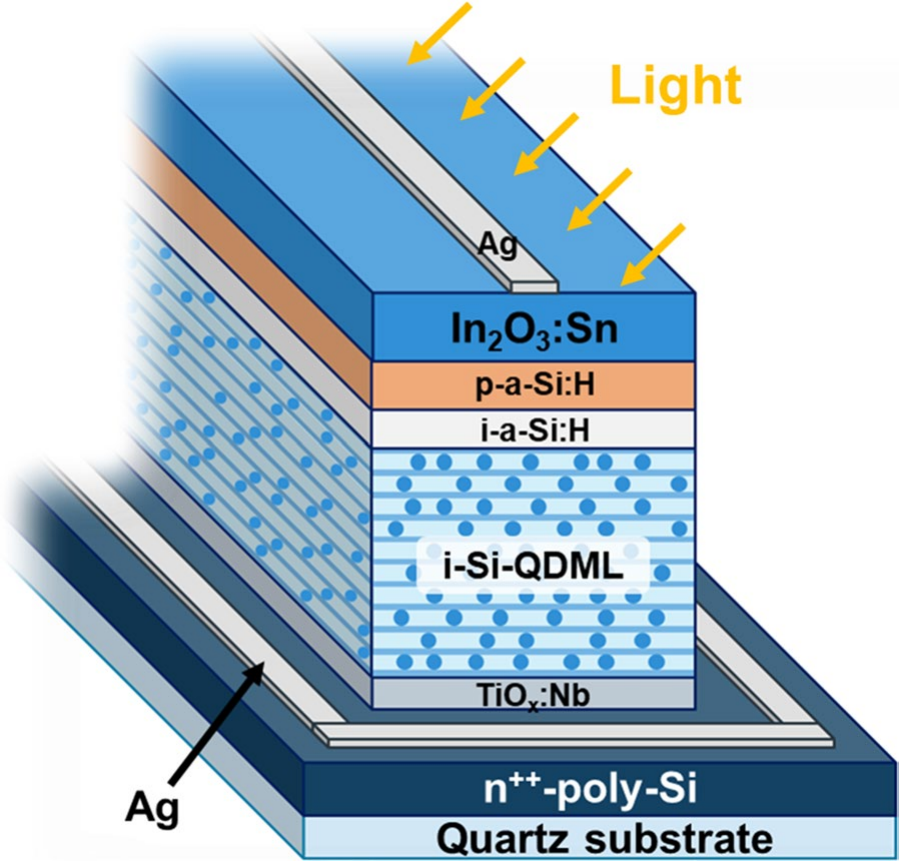
Schematic of the fabricated Si-QDSCs structure

**Fig. 4:**
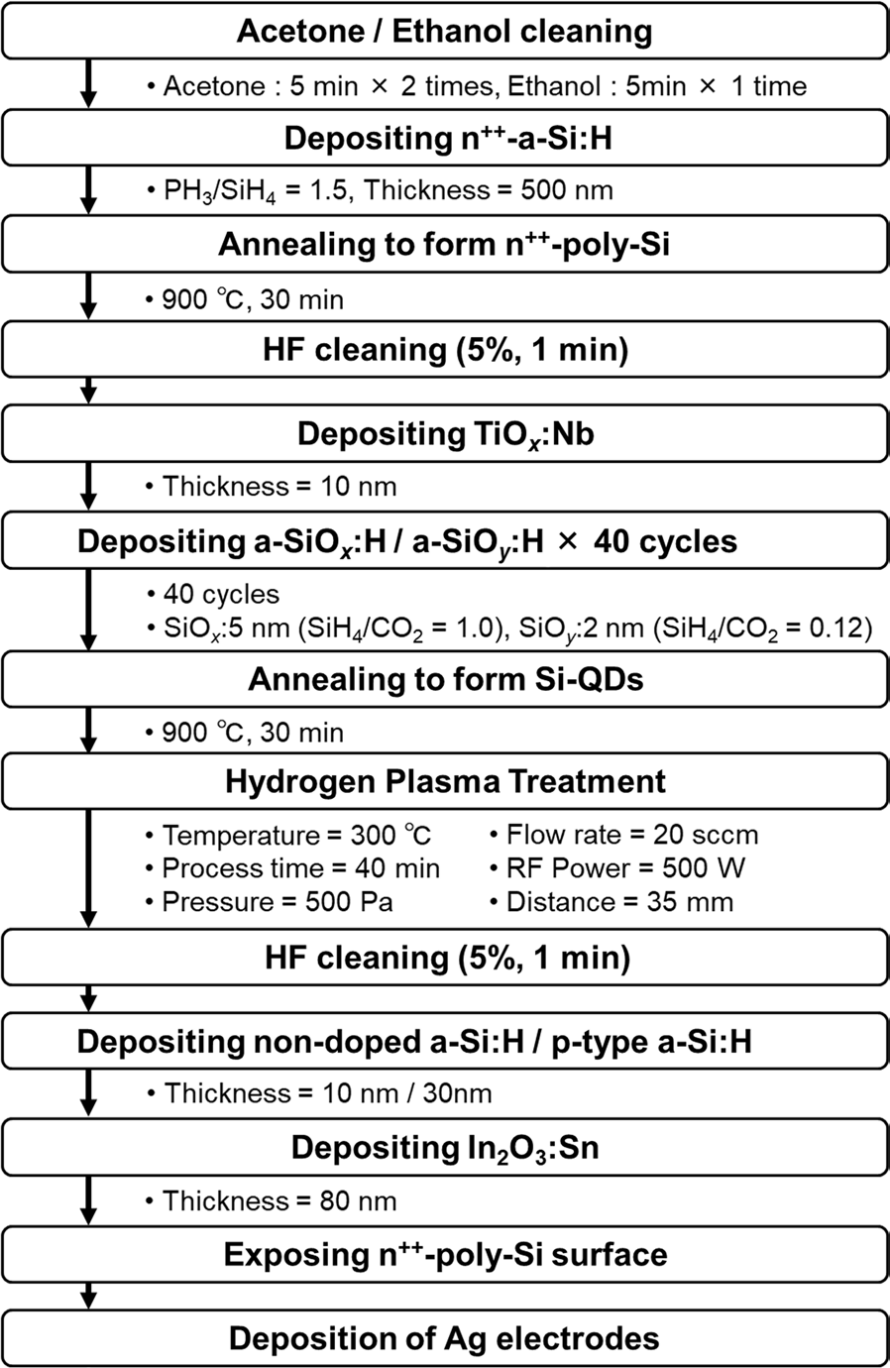
Schematic of the fabrication process of Si-QDSCs

Solar cells with Si-QDML as the absorption layer were fabricated. The fabricated solar cell structure and fabrication process are shown in Fig. 3 and Fig. 4, respectively. 500-nm-thick n^++^-a-Si:H was deposited by PECVD method at the flow rate ratio of PH_3_/SiH_4_ = 1.5 about 500 nm. The frequency was 27.12 MHz, the deposition temperature was 195 °C, the chamber pressure was 25 Pa, and the RF power was 32.5 mW/cm^2^. After deposition, n^++^-poly-Si was formed by annealing at 900 °C for 30 min under a forming gas atmosphere. Since a thermal oxide film was formed during this annealing process [17], it was removed by cleaning with 5% HF for 1 min. TNO at 10 nm thickness was deposited by RF magnetron sputtering (ULVAC, CS-200) as a diffusion barrier layer for phosphorus (P) in n^++^-poly-Si immediately after HF treatment. The deposition temperature was room temperature, argon gas flow rate and pressure were 30 sccm and 0.2 Pa, and RF power was 137 mW/cm^2^. 5-nm-thick a-SiO_*x*_:H and 2-nm-thick a-SiO_*y*_:H (*x* < *y*) layers were alternately deposited by PECVD for 40 cycles as Si-rich and barrier layers, respectively. The deposition temperature, chamber pressure, and RF power were the same as described before. Si-QDs were formed in the Si-rich layer by annealing at 900 °C for 30 min under a forming gas atmosphere. DBs on the surface of Si-QDs in the Si-QDML were reduced by introducing hydrogen atoms by HPT at 60 MHz frequency. The *T*_HPT_, *t*_HPT_, *p*_H2_, *R*_H2_, *P*_RF_, and *d* of HPT process parameters were optimized by BO. Since it was confirmed that an oxide film was formed on the surface of Si-QDML by HPT, it was removed by cleaning with 5% HF for 1 min again. 10-nm-thick non-doped a-Si:H (i-a-Si:H) layer and 30-nm-thick boron-doped a-Si:H (p-a-Si:H) layer were deposited by PECVD method. The deposition temperature, chamber pressure, and RF power were the same as Si-QDML preparation. 80-nm-thick indium tin oxide (In_2_O_3_:Sn, ITO) layer was deposited by RF magnetron sputtering. The deposition temperature, argon gas flow rate, chamber pressure, and RF power density were room temperature, 50 sccm, 0.2 Pa, and 46 mW/cm^2^, respectively. For backside electrode deposition, the surface of n^++^-poly-Si was exposed by reactive ion etching (RIE-10NR by Samco Co., Ltd.). CF_4_ was used as the etching gas at a flow rate of 20 sccm. The power was 20 W, and the process pressure was 1.0 Pa. The surface Ag electrode was fabricated by thermal evaporation and the Ag backside electrode was coated with Ag paste. Current density (*J*)–voltage (*V*) measurement was carried out under the solar simulator illumination at AM1.5G, 100 mW/cm^2^, and room temperature. Quantum efficiency was also measured at room temperature (CEP-25BX by Bunkoukeiki Co., Ltd.).

## Results and discussion

### Preparation of Si-QDML applying BO

**Fig. 5:**
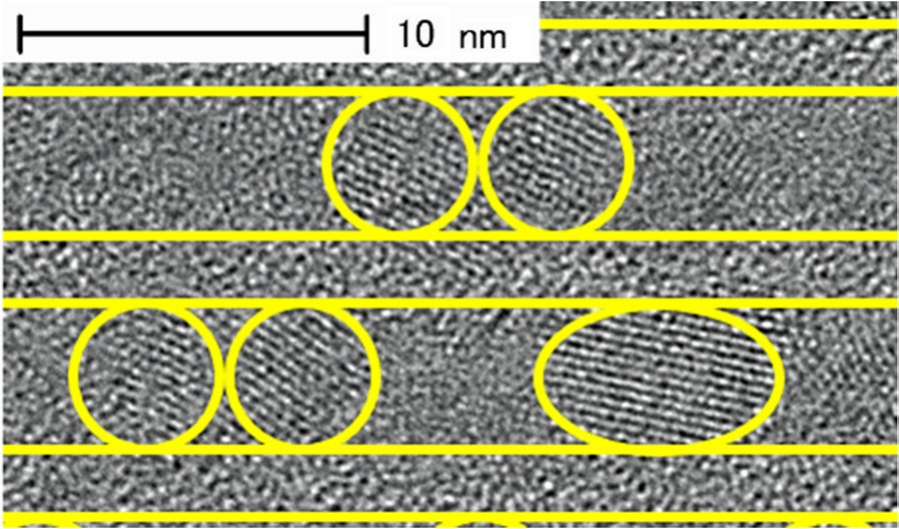
Cross-sectional TEM image of a-SiO_*x*_:H/a-SiO_*y*_:H after annealing. In this image, the existences of Si-QDs and boundaries of a-SiO_*x*_:H/a-SiO_*y*_:H are indicated using circles and straight lines, respectively

Figure 5 shows a cross-sectional TEM image of a-SiO_*x*_:H/a-SiO_*y*_:H after annealing. The fringes, originating from the Si-QDs, with the size of about 5 nm can be seen in the Si-rich layers. The size of Si-QDs roughly corresponds to the thickness of the Si-rich layer. In contrast, there is no fringes in the barrier layer. As a result, size-controlled Si-QDML was successfully obtained.

**Fig. 6:**
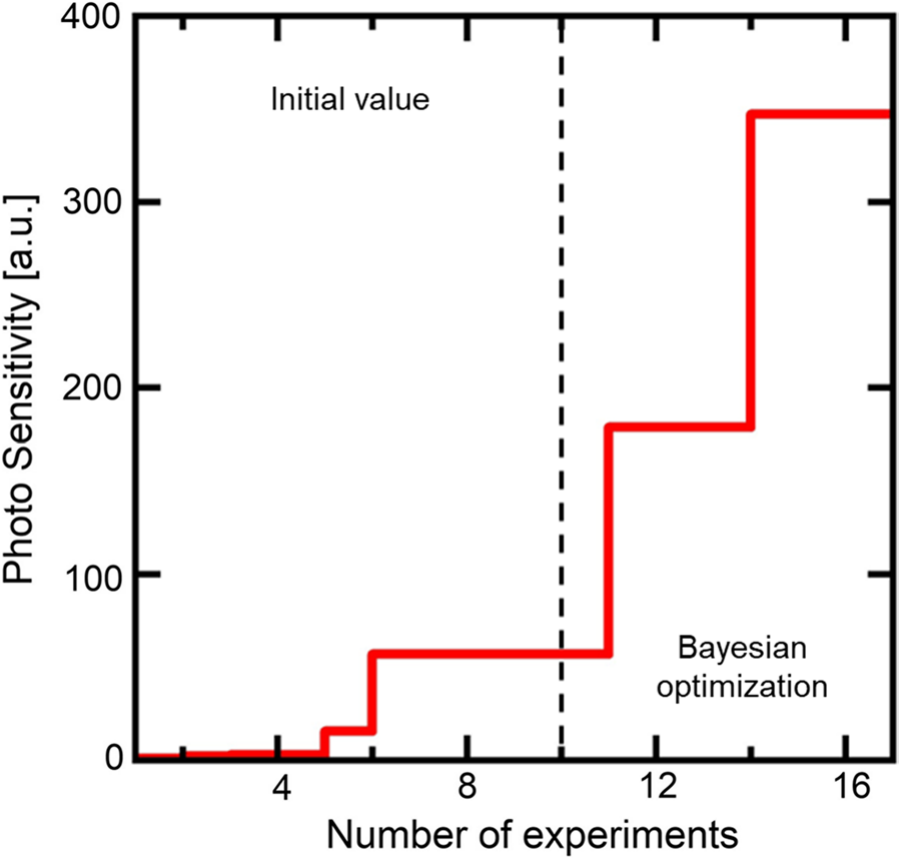
PS developments in Si-QDML. The 10 experimental cycles for the initial point and the 7 experimental cycles for optimization are separated by black dot lines. The vertical staircase graph that rises only when the PS updates its previous highest value. Since the experimental conditions calculated after the 17th experiment had been conducted, the optimization was considered complete

**Fig. 7:**
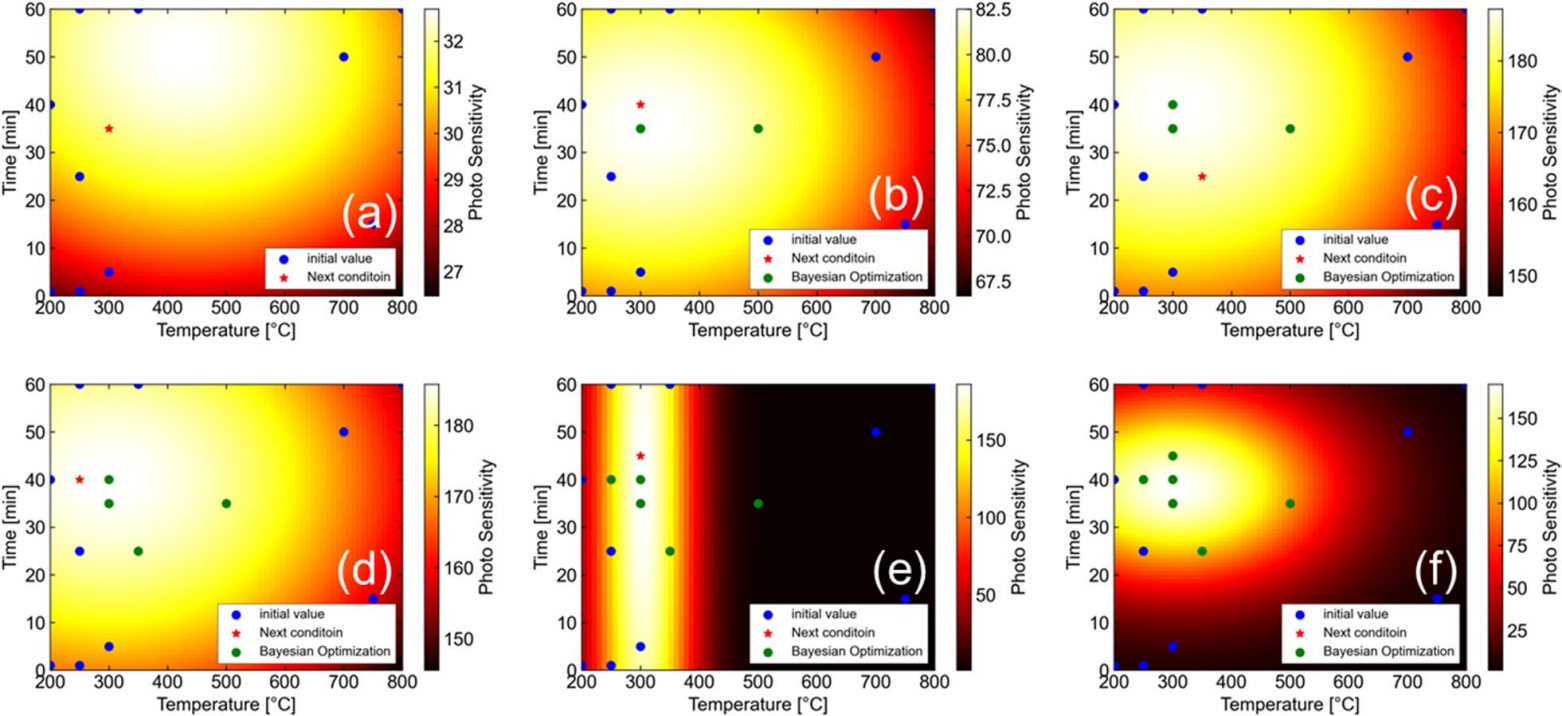
The expected value curved surfaces of the PS of Si-QDML as a function of *T*_HPT_ and *t*_HPT_. **a** Expected value curved surface with only 10 initial experiments. Then, the expected value curved surfaces at the time of **b** 3rd cycles (10 initial experiments + 3 optimization cycles), **c** 4th cycles (10 initial experiments + 4 optimization cycles), **d** 5th cycles (10 initial experiments + 5 optimization cycles), **e** 6th cycles (10 initial experiments + 6 optimization cycles), and **f** 7th cycles (10 initial experiments + 7 optimization cycles), respectively

Figure 6 shows the development of PS values of Si-QDML through the optimization of the HPT process parameters by using BO. Only when the maximum PS was updated, the step is notated to be increased. In this study, optimization of the HPT process parameters was completed through 10 times initial experiments and 7 times additional experiments based on BO (see Supplementary information 4). For example, when optimizing process parameters for a 6-parameter, 5-level process, since there are $6^{5} = 7776$ combinations of parameters, the number of experiments could be greatly reduced by using BO. Figure 7 shows the expected value curve surfaces of PS of Si-QDML. Figure 7 (a-f) is shown as a function of *T*_HPT_ and *t*_HPT_, at BO of (a) initial point only, (b) 3rd, (c) 4th, (d) 5th, (e) 6th, and (f) 7th cycles, respectively and, also, shown as the cross section from the six-dimensional space where other parameters were set to the optimized values at the cycle. Note that the actual experimental points may not exist on that cross section since the other parameters are unfixed for each experiment. The expected value curve surfaces became steeper with increasing the number of experimental cycles, especially after the 6th cycle. It suggests that the parameters were rapidly converged to one specific region. Finally, after the 8th cycle of UCB calculations, the next experimental parameters were the same as the HPT process parameters at the highest PS in the previous cycle. At this time, *T*_HPT_ and *t*_HPT_ were converged to 300 °C and 40 min. From the BO perspective, the optimal region obtained in this case is considered reasonable since the expected value curve surfaces in parameter space should be smooth with no singularities physically. From the HPT perspective, the study on SiC reported that the *T*_HPT_ of 300 °C was the optimal temperature to reduce Si-DBs [6] and it is consistent with this optimal results by using BO. It is speculated that the reason for the lower expected value curve surfaces at lower temperatures is that hydrogen diffusion in the Si-QDML was insufficient to terminate DBs located deeply in the sample. On the other hand, the reason for the lower expected value curve surfaces at higher temperatures may be that hydrogen atoms were desorbed from the Si–H bonding state at temperatures above 350 °C, leading to the reduction of the effect of dangling bond termination. This temperature range corresponds to the temperature of hydrogen desorption of the Si–H bonding state [43].

**Fig. 8:**
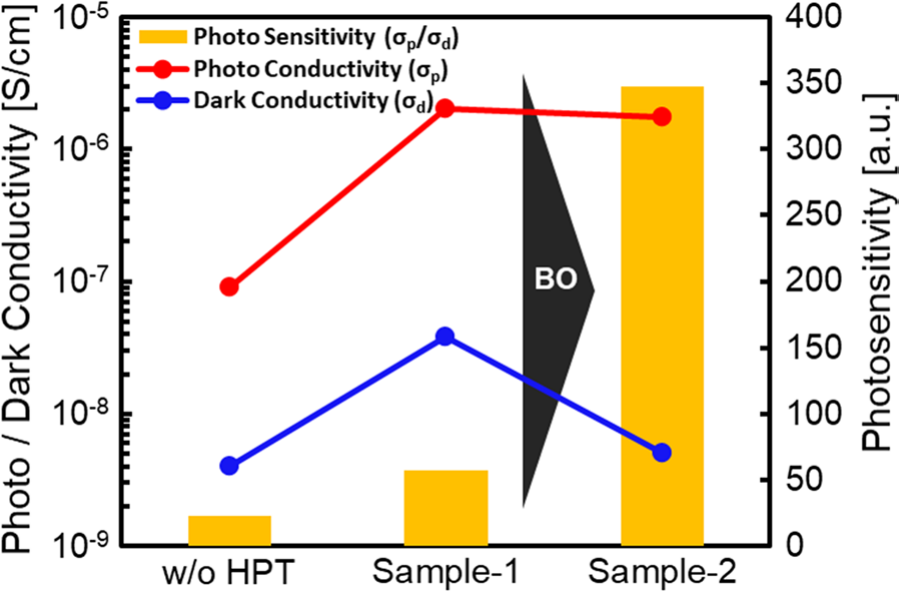
*σ*_p_, *σ*_d_ and PS in the sample w/o HPT, Sample-1 (sample with the highest PS at BO initial point acquisition), and Sample-2 (sample after optimization by BO)

Table 2 shows HPT process parameters, *σ*_p_, *σ*_d_ and PS of w/o HPT sample, Sample-1 (sample with the highest PS at BO initial point acquisition), and Sample-2 (sample after optimization by BO). Figure 8 shows *σ*_p_, *σ*_d_ and PS of w/o HPT sample, Sample-1, and Sample-2. Thanks to the BO, the PS was clearly improved from 22.7 to 347.2. Generally, the physical properties of device-grade hydrogenated microcrystalline silicon (μc-Si:H) are $\sigma_{{\text{p}}} = 10^{ - 4} - 10^{ - 3}$ S/cm, $\sigma_{{\text{d}}} = 10^{ - 7} - 10^{ - 5}$ S/cm, and ${\text{PS}} = 10^{1} - 10^{3}$ [44]. The Sample-2 has a comparable electrical properties with the μc-Si:H. Since the highest efficiency of 11.9% has been reported for single-junction solar cells using μc-Si:H [45, 46], it is expected that the efficiency of solar cells with the Sample-2 is improved. Comparing the conductivity w/o HPT and Sample-1, the significant increase in *σ*_p_ suggests that the DBs were terminated and *τ* (Eqs. (2) and (4)) increased.

**Table 2:** HPT process parameters and electrical characteristics in the sample w/o HPT and Sample-1 (sample with highest PS at BO initial point acquisition) and Sample-2 (sample with hydrogen termination at HPT process parameters after BO)

	*T*_HPT_ (°C)	*t*_HPT_ (min)	*p*_H2_ (Pa)	*R*_H2_ (sccm)	*P*_RF_ (W)	*d* (mm)	*σ*_p_ (S/cm)	*σ*_d_ (S/cm)	PS (–)
w/o HPT	–	–	–	–	–	–	9.1 × 10^–8^	4.0 × 10^–9^	22.7
Sample-1	350	60	400	20	500	45	2.0 × 10^–6^	3.8 × 10^–8^	57.1
Sample-2	300	40	500	20	500	35	1.8 × 10^–6^	5.1 × 10^–9^	347.2

**Fig. 9:**
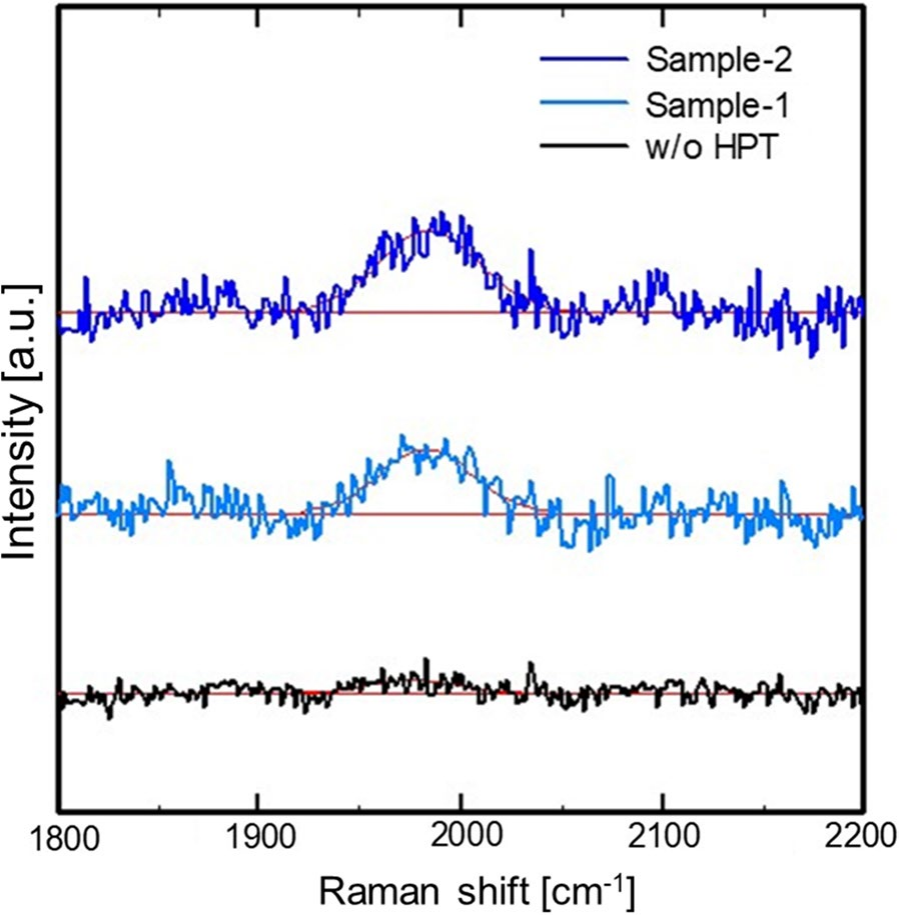
Raman scattering spectra around 2000 cm^−1^ of the sample w/o HPT, Sample-1 and Sample-2

Figure 9 shows Raman scattering spectra around 2000 cm^−1^ of w/o HPT sample, Sample-1, and Sample-2. In the case of w/o HPT sample, there is no band around 2000 cm^−1^. It is due to the desorption of hydrogen in the Si-QDML by annealing to form Si-QDs. On the other hand, the broad band appeared around 2000 cm^−1^ in Sample-1 and Sample-2. The band around 2000 cm^−1^ corresponds to a Si–H stretching mode [47, 48]. It suggests that hydrogen was introduced into the Si-QDML by HPT and DBs were terminated by hydrogen. The Raman scattering measurements confirmed that the increase in *σ*_p_ shown in Fig. 8 was due to the hydrogen termination of DBs by HPT, resulting in an increase in *τ*.

#### Fabrication of solar cell structure

**Fig. 10:**
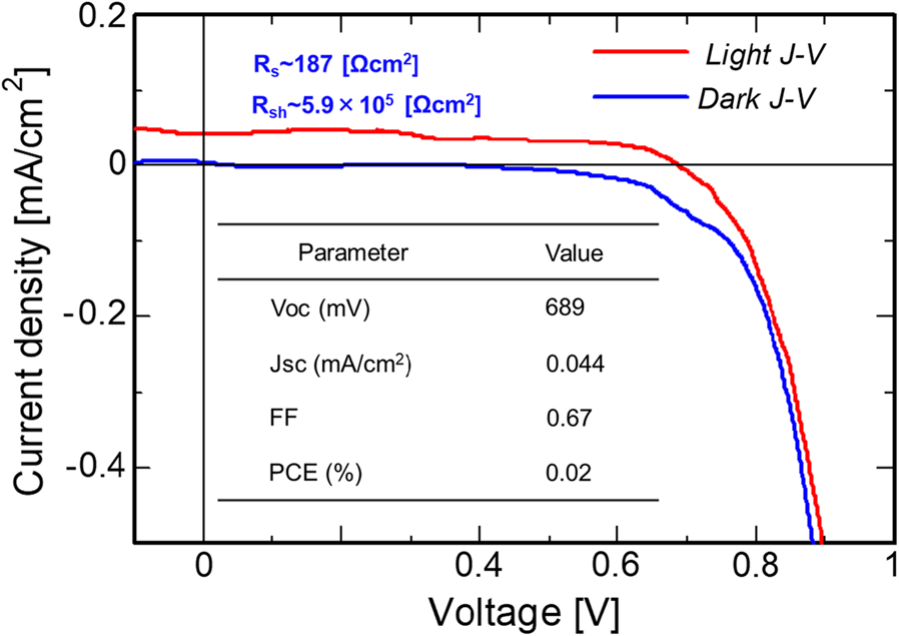
Dark and illuminated *J*–*V* characteristics of the fabricated Si-QDSC

Figure 10 shows the dark and light *J*–*V* characteristics of the Si-QDSCs. Note that Si-QDML was prepared through the same process parameters as Sample-2. The solar cell parameters obtained from the light *J*–*V* characteristics are also summarized in Fig. 10. The *J*-*V* curve of the solar cell showed a rectifying property. Under AM1.5G illumination, the photovoltaic effect was obtained. From the obtained *J*–*V* characteristics, the diode quality factor and the reverse saturation current density of the solar cell were 1.23 and $1.41 \times 10^{ - 11}$ mA/cm^2^, respectively. In particular, extremely high values of 689 mV and 0.67 were obtained for *V*_OC_ and fill factor (FF), respectively. These *V*_OC_ and FF are the highest values for this type of solar cells, which were achieved through an unprecedented attempt to combine HPT and BO. The increase in *V*_OC_ and FF is due to the improvement of the film quality of Si-QDML. In the case of p-i-n-type solar cells, the dangling bonds in the i-layer degrade the built-in potential, leading to the degradation of *V*_OC_. Guha et al. simulated the amorphous silicon solar cell performance by using AMPS-1D program [49]. The simulation results showed that *V*_OC_, *J*_SC_, and FF decrease with the increase of defect density in the i-a-Si:H layer. The expected decrease in *V*_OC_ as the defect density changes is calculated from the changes in the quasi-Fermi-levels for electrons and holes [50]. The corresponding decline in the photoconductivity of the i-a-Si:H films is roughly proportional to the reciprocal defect density [51]. Therefore, the reduction of dangling bonds in the Si-QDML leads to the improvement of *V*_OC_. The degradation of FF is due to the field distortion by space charge derived from the defects in the a-Si:H layer [52]. *J*_SC_ was also significantly improved from 0.02 to 0.044 mA/cm^2^ compared to the solar cell fabricated by Perez-Wurfl et al. [30]. Compared to them, the increases in *V*_OC_ and *J*_SC_ were also caused by a clear improvement in FF, which is due to a decrease in series resistance (*R*_s_) and an increase in shunt resistance (*R*_sh_). The increase in *J*_SC_ was attributed to a significant reduction of *R*_s_ from 4.1 × 10^3^ to 187 Ω･cm^2^. This is due to the enhancement of tunneling probability by using a-SiO_*y*_ barrier layer instead of SiO_2_, leading to the reduction of *R*_s_ in the absorption layer. A noteworthy high value of $R_{{{\text{sh}}}} = 5.9 \times 10^{5}$ Ω･cm^2^ was also confirmed. This result indicates that the fabricated solar cells have high isolation qualities and low defects in the absorption layer due to HPT by using BO. Further improvement of PCE requires improvement of *J*_SC_. The insufficient *J*_SC_ was due to the diffusion of dopant atoms during device fabrication, resulting in a thinner effective thickness of the photoactive layer. The solution is increasing the thickness of the Si-QDML, or adopting the “Dopant-grading” proposed by Pham et al. to control the diffusion of P into the absorption layer [53]. Otherwise, the implementation of the optical confinement structure is expected to increase the optical path length in the absorption layer and improve *J*_SC_.

**Fig. 11:**
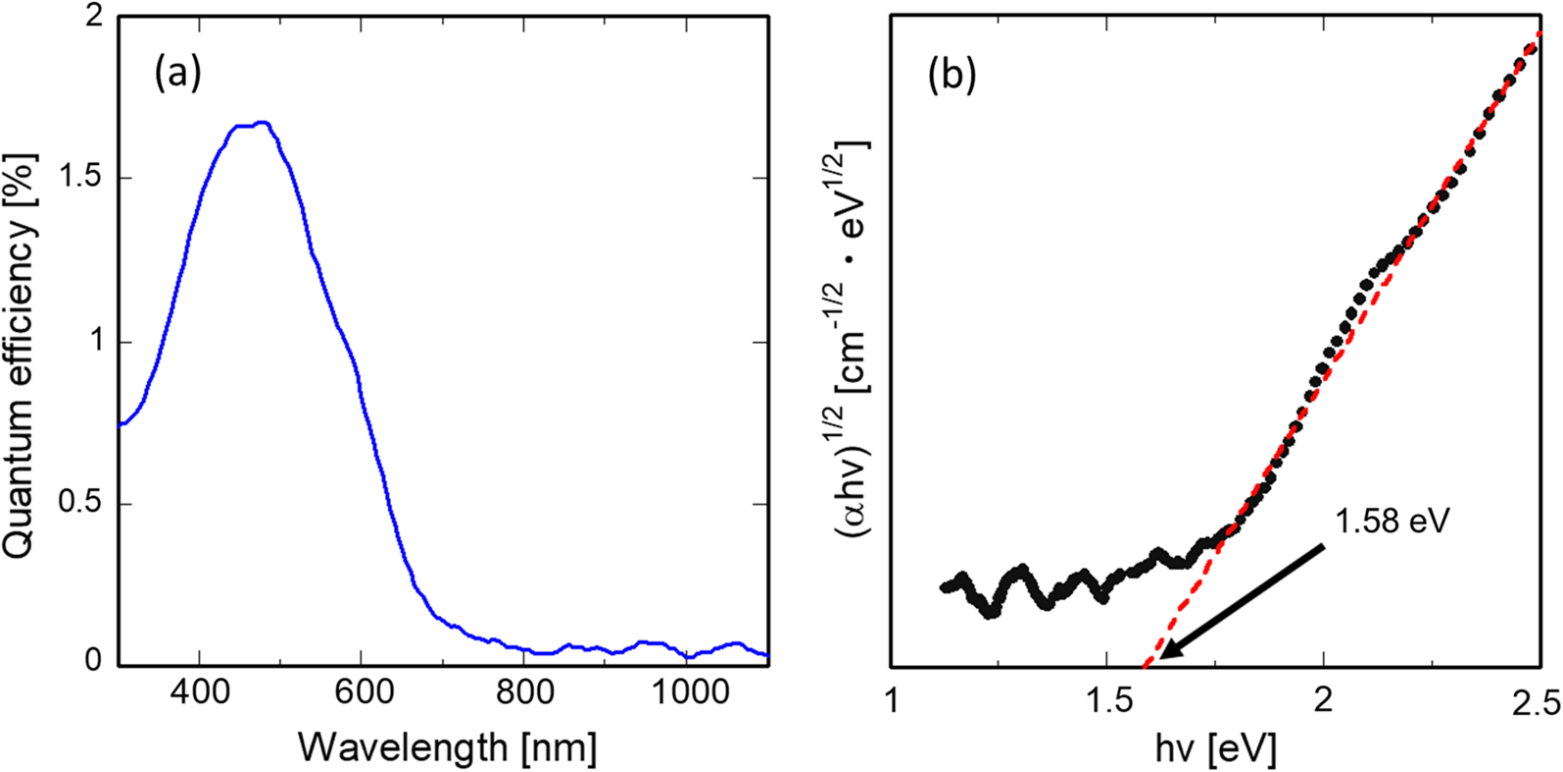
**a** External quantum efficiency of the fabricated Si-QDSC. **b** Tauc plot calculated from the EQE data

Figure 11 (a) shows the EQE of a solar cell with Si-QDML as the absorption layer. The quantum efficiency shows a clear peak in the range below about 800 nm. The absorption edge is located around 800 nm. This wavelength corresponds to the bandgap of Si-QDML [1], suggesting that the carrier generation was contributed by the Si-QDML rather than n^++^-poly-Si, which was heavily doped and very low minority carrier lifetime due to Auger recombination. To estimate the absorption wavelength edge, the absorption coefficient *α* was roughly estimated. When normal incidence, no back reflection, and no carrier recombination were assumed, ${\text{EQE}}\left( \lambda \right)$ for each wavelength $\lambda$ can be calculated from reflectance $R\left( \lambda \right)$, absorption coefficient $\alpha \left( \lambda \right)$, and absorption layer thickness *d*_ab_ according to Lambert–Beer’s law as follows.

7 $$\begin{array}{*{20}c} {{\text{EQE}}\left( \lambda \right) = \left( {1 - R\left( \lambda \right)} \right)\left[ {1 - e^{{ - \alpha \left( \lambda \right)d_{{{\text{ab}}}} }} } \right]} \\ \end{array}$$

Transforming Eq. 7 into an equation for $\alpha \left( \lambda \right)$ leads to:

8 $$\begin{array}{*{20}c} {\alpha \left( \lambda \right) = - \frac{1}{{d_{{{\text{ab}}}} }}\ln \left( {1 - \frac{{{\text{EQE}}\left( \lambda \right)}}{1 - R\left( \lambda \right)}} \right)} \\ \end{array}$$

In general, the absorption coefficient α can be written as a function of the incident photon energy *hν*,

9 $$\begin{array}{*{20}c} {\alpha \left( \lambda \right) = A\frac{{\left( {h\nu - E_{{\text{g}}} } \right)^{{n_{{\text{c}}} + n_{{\text{v}}} + 1}} }}{h\nu }} \\ \end{array}$$

where *A* is the proportionality constant, $E_{{\text{g}}}$ is the corresponding bandgap, $n_{{\text{c}}} = \left( {D_{{\text{c}}} - 2} \right)/2$ and $n_{{\text{v}}} = \left( {D_{{\text{v}}} - 2} \right)/2$. Here, $D_{{\text{c}}}$ and $D_{{\text{v}}}$ are the fractal dimensionalities of the conduction and valence bands, respectively, and assuming that both are parabolic, $n_{{\text{c}}} = n_{{\text{v}}} = 1/2$ for 3D space [54]. Equation 9 leads to

10 $$\alpha \left( \lambda \right) = A\frac{{\left( {h\nu - E_{{\text{g}}} } \right)^{2} }}{h\nu }$$

11 $$\left( {\alpha \left( {h\nu } \right) \cdot h\nu } \right)^{1/2} = A^{\prime}\left( {h\nu - E_{{\text{g}}} } \right)$$

where *A'* is the proportionality constant. Figure 11 (b) shows a Tauc plot with $\left( {\alpha h\nu } \right)^{1/2}$ calculated from the absorption coefficient $\alpha \left( \lambda \right)$ which is obtained from Eq. 8 using the measured ${\text{EQE}}\left( \lambda \right)$ and reflectance. The bandgap was estimated from the Tauc plot to be about 1.58 eV. Since this bandgap is obtained from EQE, it is called “effective mobility gap.” In the case of a typical a-Si:H thin films, the effective mobility gap is higher than the optical gap. Wronski et al. reported that the effective mobility gap at room temperature in intrinsic a-Si:H having a Tauc optical gap of 1.73 eV is 1.89 ± 0.03 eV [55]. From our measurement, the optical bandgaps of a-SiO_*x*_:H single layer, a-SiO_*y*_:H single layer, and a-SiO_*x*_:H/a-SiO_*y*_:H multilayer were 2.04, 1.95, and 2.43 eV, respectively. From these results, the effective mobility gap of these amorphous silicon oxide thin films should be more than about 2 eV, which is far from the estimated value of 1.58 eV. Therefore, we concluded the estimated bandgap of 1.58 eV is derived from the carrier paths due to Si-QDs.

## Conclusion

In summary, first, the Si-QDML was prepared by depositing a-SiO_*x*_:H/a-SiO_*y*_:H (*x* < *y*) and annealing at 900 °C. The cross-sectional TEM image confirmed the striped Si-QDML structure and the formation of Si-QDs in the Si-rich layer corresponding to the layer thickness. Secondly, the HPT process parameters for Si-QDML were optimized by using BO, since the HPT has many parameters such as *T*_HPT_ and *t*_HPT_, and it is difficult to optimize the parameters by exhaustive experiments. So, the parameters were efficiently optimized based on probabilistic expectations by using BO. By repeating calculations and experiments, the six-dimensional parameter spaces were explored globally in only 17 experimental cycles (including 10 experimental cycles at the initial point), and PS, an important electrical characteristic in solar cells, was successfully improved. Since the next experimental parameters calculated by the UCB were identical to the results of previous calculations, the optimization was completed. The optimized HPT process parameters were: $T_{{{\text{HPT}}}} = 300$ °C, $t_{{{\text{HPT}}}} = 40$ min, $p_{{{\text{H}}_{2} }} = 500$ Pa, $R_{{{\text{H}}_{2} }} = 20$ sccm, $P_{{{\text{RF}}}} = 500$ W, $d = 35$ mm when ${\text{PS}} = 347$ was confirmed. Physically, this improvement in PS is attributed to the simultaneous achievement of two factors: 1. increased carrier lifetime due to hydrogen termination of DBs and 2. suppression of thermal donor formation, which was a side effect of the HPT perspective. Finally, Si-QDSCs were fabricated. Note that HPT was performed on Si-QDs using optimized HPT process parameters. *V*_OC_ and FF of 689 mV and 0.67, respectively, were achieved. In the measured EQE, a clear peak was observed at the range below about 800 nm and the estimated band gap was about 1.58 eV. This suggests that Si-QDML contributed to the carrier generation. Through this study, we were able to demonstrate the usefulness of HPT in solar cells with Si-QDML as the absorption layer and the usefulness of BO in a real experimental process with multiple parameters. In addition, the optimized HPT process parameters can contribute to the higher efficiency of Si-QDSCs.

## Supplementary Information

Supplementary Information

## Data Availability

All data supporting the conclusions of this article are included within the article.

## Abbreviations

Si-QD: Silicon quantum dot
SiNW: Silicon nanowire
c-Si: Crystalline silicon
a-SiC: Amorphous silicon carbide
SiO_2_: Silicon dioxide
Si-QDSC: Silicon quantum dot solar cell
Si-QDML: Silicon quantum dot multilayer
*V*_OC_: Open-circuit voltage
*J*_SC_: Short-circuit current
a-SiCO_*x*_: Amorphous silicon oxycarbide
a-SiO_*y*_: O-deficient amorphous silicon oxide
DB: Dangling bond
HPT: Hydrogen plasma treatment
BO: Bayesian optimization
PS: Photosensitivity
*σ*_p_: Photoconductivity
*σ*_d_: Dark conductivity
TiO_*x*_:Nb, TNO: Niobium-doped titanium oxide
n^++^-poly-Si: N-type polycrystalline silicon
a-SiO_*x*_:H/a-SiO_*y*_:H (*x* < *y*): Hydrogenated amorphous silicon oxide multilayers
PECVD: Plasma-enhanced chemical vapor deposition
*T*_HPT_: Process temperature of HPT
*t*_HPT_: Process time of HPT
*p*_H2_: H_2_ pressure of HPT
*R*_H2_: H_2_ flow rate of HPT
*P*_RF_: RF power of HPT
*d*: Electrode distance of HPT
GPR: Gaussian process regression
UCB: Upper confidence bound
P: Phosphorus
i-a-Si:H: Non-doped a-Si:H
p-a-Si:H: Boron-doped a-Si:H
In_2_O_3_:Sn, ITO: Indium tin oxide
μc-Si:H: Microcrystalline silicon
FF: Fill factor
*R*_s_: Series resistance
*R*_sh_: Shunt resistance
