# Supplementary material for: Bayesian optimization of hydrogen plasma treatment in silicon quantum dot multilayer and application to solar cells
Source: Discov Nano. 2023 Mar 13;18(1):43. doi: 10.1186/s11671-023-03821-9 (PMC10214912; doi:10.1186/s11671-023-03821-9)
Supplement: Supplementary file 1 — Supplementary Information [file 11671_2023_3821_MOESM1_ESM.docx]

**Supplementary Information (SI)**

**Bayesian optimization of hydrogen plasma treatment in silicon quantum dot multilayer and application to solar cells**

**Fuga Kumagai ^1^*, *Kazuhiro Gotoh ^1^*, *Satoru Miyamoto ^1^*, *Shinya Kato ^2^*,

*Kentaro Kutsukake ^3^*, *Noritaka Usami ^1^*, *Yasuyoshi Kurokawa ^1^*

1: Materials Process Engineering, Graduate School of Engineering, Nagoya University, Furo-cho, Chikusa-ku, Nagoya, 464-8603, Japan.

2: Department of Electrical and Mechanical Engineering, Nagoya Institute of Technology, Showa-ku, Nagoya, 466-8555, Japan.

3: Center for Advanced Intelligence Project, RIKEN, Tokyo, 103-0027, Japan.

*E-mail: [kumagai.fuga.v1@s.mail.nagoya-u.ac.jp](mailto:kumagai.fuga.v1@s.mail.nagoya-u.ac.jp),
 [kurokawa.yasuyoshi@material.nagoya-u.ac.jp](mailto:kurokawa.yasuyoshi@material.nagoya-u.ac.jp)


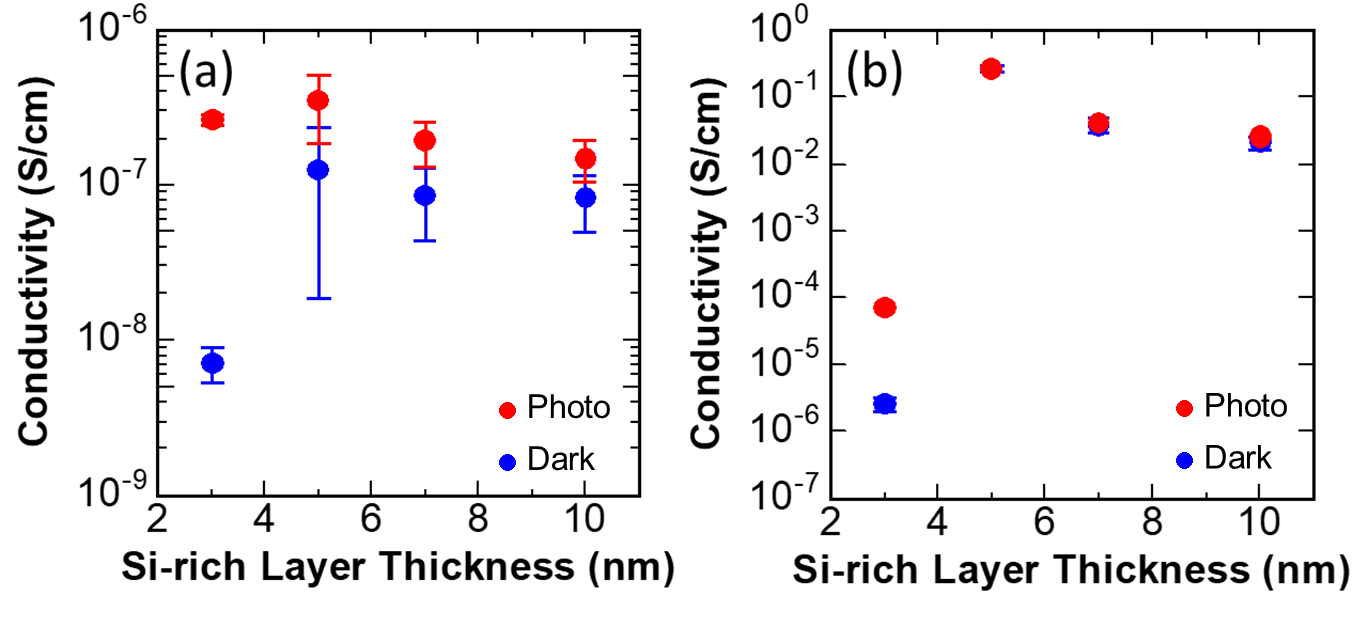
Figure SI-1 | *σ*_p_ and *σ*_d_ of Si-QDMLs (a) before and (b) after HPT. Si-QDMLs with the target diameter of $t=3, 5, 7, \mathrm{and} 10 \mathrm{nm}$ were prepared by PECVD of SiO*_x_*(Si-rich layer: $t$nm)/SiO*_y_*(2 nm) multilayer (*x*<*y*) and post-annealing at 900 ^o^C. HPT was performed on the samples, at process temperature, process time, H_2_ pressure, H_2_ flow rate, RF power, and electrode distance of 800 °C, 60 min, 400 Pa, 200 sccm, 450 W and 50 mm, respectively. In the case of this HPT condition, the conductivity after HPT was increased by about 5-6 orders of magnitude compared to that before HPT when *t* was more than 5 nm, leading to small PS. It is speculated that the carrier density in the Si-QDMLs was increased drastically by HPT. Since the increase in the carrier density by HPT is much larger than the photogenerated carrier density by the illumination of AM1.5G. That is why *σ*_p_ was comparative to *σ*_d_. A thermal donor is one possibility to explain the drastical increase in *σ*_d_. Thermal donors are negated when Czochralski silicon is neated at a temperature in the range of 300-500 ^o^C [S1]. Murray *et al.* reported that the formation of thermal donors in Czochralski silicon was promoted by HPT due to oxygen diffusion [S2]. Therefore, HPT at high temperatures such as 800 ^o^C results in the formation of oxygen defects in the Si-QDMLs, which is the cause of thermal donor formation and PS decrease. In this study, the formation of thermal donors in the Si-QDMLs was suppressed by a lower *T*_HPT_ at 300 ^o^C, which is the optimized *T*_HPT_, high PS was obtained.


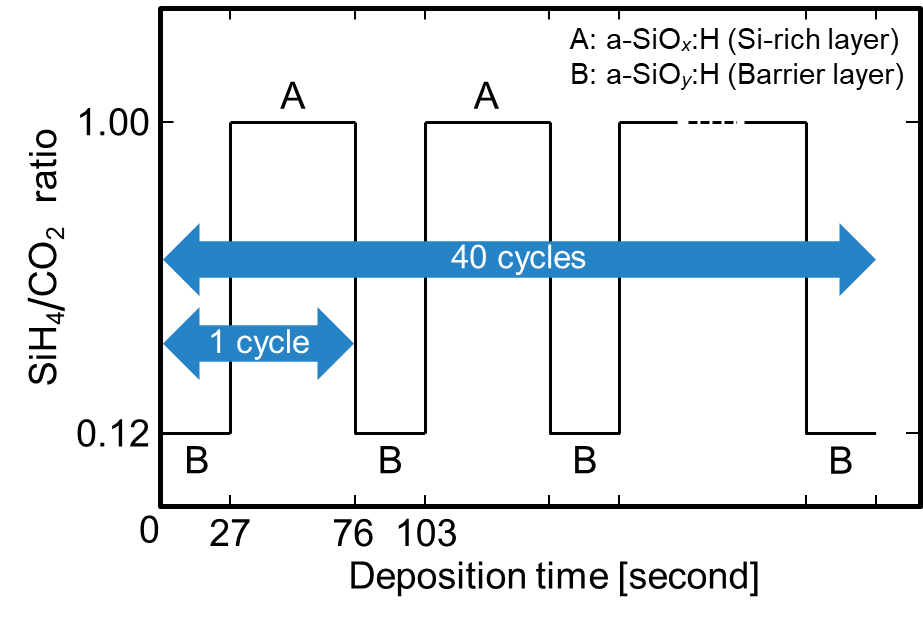


Figure SI-2 | SiH_4_/CO_2_ flow rate ratio at each deposition time during the deposition of a-SiO*_x_*:H/a-SiO*_y_*:H multilayers. The SiH_4_/CO_2_ ratios of the Si-rich and barrier layers were 1.0 and 0.12, respectively. The deposition times for each layer in a-SiO*_x_*:H/a-SiO*_y_*:H (*x*<*y*) multilayers were 49 and 27 s to obtain 5-nm-thick a-SiO*_x_*:H and 2-nm-thick a-SiO*_y_*:H, respectively. The period was 40 cycles.

Table SI-3 | Optical bandgaps, *σ*_p_, *σ*_d_ and PS of a-SiO*_x_*:H layer, a-SiO*_y_*:H layer, and a-SiO*_x_*:H/a-SiO*_y_*:H (*x*<*y*) multilayer. The optical gaps were estimated by spectroscopic ellipsometry. Tauc-Lorentz model was used as a fitting function. These *σ*_p_ and *σ*_d_ were measured as same as those of Si-QDML.

|  | Optical bandgap  [eV] | *σ*_p_  [S/cm] | *σ*_d_  [S/cm] | PS  [-] |
| --- | --- | --- | --- | --- |
| a-SiO*_x_*:H  (Si-rich layer) | 1.95 | 9.6×10^-8^ | 1.2×10^-10^ | 777.9 |
| a-SiO*_y_*:H  (Barrier layer) | 2.43 | 8.4×10^-10^ | 7.5×10^-11^ | 11.3 |
| a-SiO*_x_*:H/a-SiO*_y_*:H(*x*<*y*)  multilayer | 2.04 | 8.0×10^-9^ | 7.8×10^-11^ | 102.9 |

Table SI-4 | HPT conditions for 10 times initial experiments and 7 times additional experiments based on the Bayesian optimization process.

|  | Number of  experiments | *T*_HPT_  [℃] | *t*_HPT_  [min] | *P*_H2_  [Pa] | *R*_H2_  [sccm] | *P*_RF_  [W] | *d*  [mm] |
| --- | --- | --- | --- | --- | --- | --- | --- |
| 10 times initial experiments | 1 | 800 | 60 | 400 | 200 | 450 | 50 |
|  | 2 | 250 | 1 | 500 | 60 | 340 | 60 |
|  | 3 | 200 | 40 | 700 | 180 | 400 | 30 |
|  | 4 | 200 | 1 | 500 | 80 | 300 | 20 |
|  | 5 | 700 | 50 | 400 | 180 | 280 | 25 |
|  | 6 | 350 | 60 | 400 | 20 | 500 | 45 |
|  | 7 | 750 | 15 | 700 | 180 | 480 | 25 |
|  | 8 | 250 | 25 | 200 | 160 | 320 | 65 |
|  | 9 | 300 | 5 | 200 | 140 | 440 | 65 |
|  | 10 | 250 | 60 | 600 | 80 | 440 | 60 |
| 7 times additional experiments | 11 | 300 | 35 | 500 | 20 | 500 | 30 |
|  | 12 | 500 | 35 | 400 | 20 | 500 | 10 |
|  | 13 | 300 | 35 | 500 | 20 | 500 | 25 |
|  | 14 | 300 | 40 | 500 | 20 | 500 | 35 |
|  | 15 | 350 | 25 | 600 | 20 | 500 | 35 |
|  | 16 | 250 | 40 | 500 | 40 | 480 | 35 |
|  | 17 | 300 | 45 | 500 | 20 | 500 | 35 |

**References**

[S1] Kaiser, W.;Frisch, H. L.; Reiss, H.: **Mechanism of the Formation of Donor States in Heat-Treated Silicon.** *Phys. Rev.* 1958, ***112***: 1546-1554.

[S2] Murray, R.;Brown, A. R.; Newman, R. C.: **Enhanced thermal donor formation and oxygen diffusion in silicon exposed to a hydrogen plasma.** *Materials Science and Engineering: B* 1989, ***4***: 299-302.
